# Supplementary material for: One-Step Genetic Modification by Embryonic Doral Aorta Injection of Adenoviral CRISPR/Cas9 Vector in Chicken
Source: Int J Mol Sci. 2024 Aug 9;25(16):8692. doi: 10.3390/ijms25168692 (PMC11354862; doi:10.3390/ijms25168692)
Supplement: Supplementary file 1 [file ijms-25-08692-s001.zip › ijms-3113589-supplementary.pdf]

SUPPLEMENTARY MATERIAL

Table S1. Oligonucleotide and primers used in this study.

Name	Forward[a]	Reverse[a]
pX459-E1-sgRNA1[b]	caccgCGGCTCCTTATATATGTTCC	aaacGGAACATATATAAGGAGCCGc
pX459-E1- sgRNA2[b]	caccgCGGCTCATGATGAGGCACAC	aaacGTGTGCCTCATCATGAGCCGc
pX459-E1- sgRNA3[b]	caccgCTGCGCCATACGCCGCGCTA	aaacTAGCGCGGCGTATGGCGCAGc
pX459-E1- sgRNA4[b]	caccgGGTCGATCGGAAGGGATCCG	aaacCGGATCCCTTCCGATCGACCc
pX459-E2- sgRNA1[b]	caccgAGTACGTCCTGCCGGCATCC	aaacGGATGCCGGCAGGACGTACTc
pX459-E2- sgRNA2[b]	caccgTCCAGGTTGCGCCGGGATGC	aaacGCATCCCGGCGCAACCTGGAc
pX459-E2- sgRNA3[b]	caccgAGAATGAGGAGCGTTGCCTG	aaacCAGGCAACGCTCCTCATTCTc
pX459-E2- sgRNA4[b]	caccgTTGCCTGCGGGACCTGGTGC	aaacGCACCAGGTCCCGCAGGCAAc
pX459 sequencing	GAGGGCCTATTTCCCATGATT	
E1-sgRNA1[c]	GGAAGTGTTTGCTGTGGGTT	CAGGTCGCAGACACTCCG
E1-sgRNA2[c]	TGGAAATGTTGGAAGCCATTCCT	GCCCAGGTCGCAGACACTC
E1-sgRNA3[c]	TGGCTTCTCCTCAGCCTCC	TTCCGATCGACCTCCTCCAA
E1-sgRNA4[c]	GGCAGAGGATTTCCTTCGGA	GATCTGCTCCTTCTCGTGGG
E2-sgRNA1[c]	CAAGCAGAGGAACCAAAGTGC	ATGCTCTGAAAAGCAACCAGC
E2-sgRNA2[c]	CAAGCAGAGGAACCAAAGTGC	ATGCTCTGAAAAGCAACCAGC
E2-sgRNA3[c]	CTCTTCCTTCTGCAGGTCCG	GTGTTGGGTGTGCAGCAATG
E2-sgRNA4[c]	CTCTTCCTTCTGCAGGTCCG	GTGTTGGGTGTGCAGCAATG
Novaseq[c]	CAAGCAGAGGAACCAAAGTGC	ATGCTCTGAAAAGCAACCAGC
G1	GCTGCTGAGAAATCAGCCCT	GTGTTGGGTGTGCAGCAATG

[a] All primers are written 5’ to 3’.

[b] sgRNA is in uppercase.

[c] Primer should be synthetized with different illumina recommended barcode added on the 5 'end.

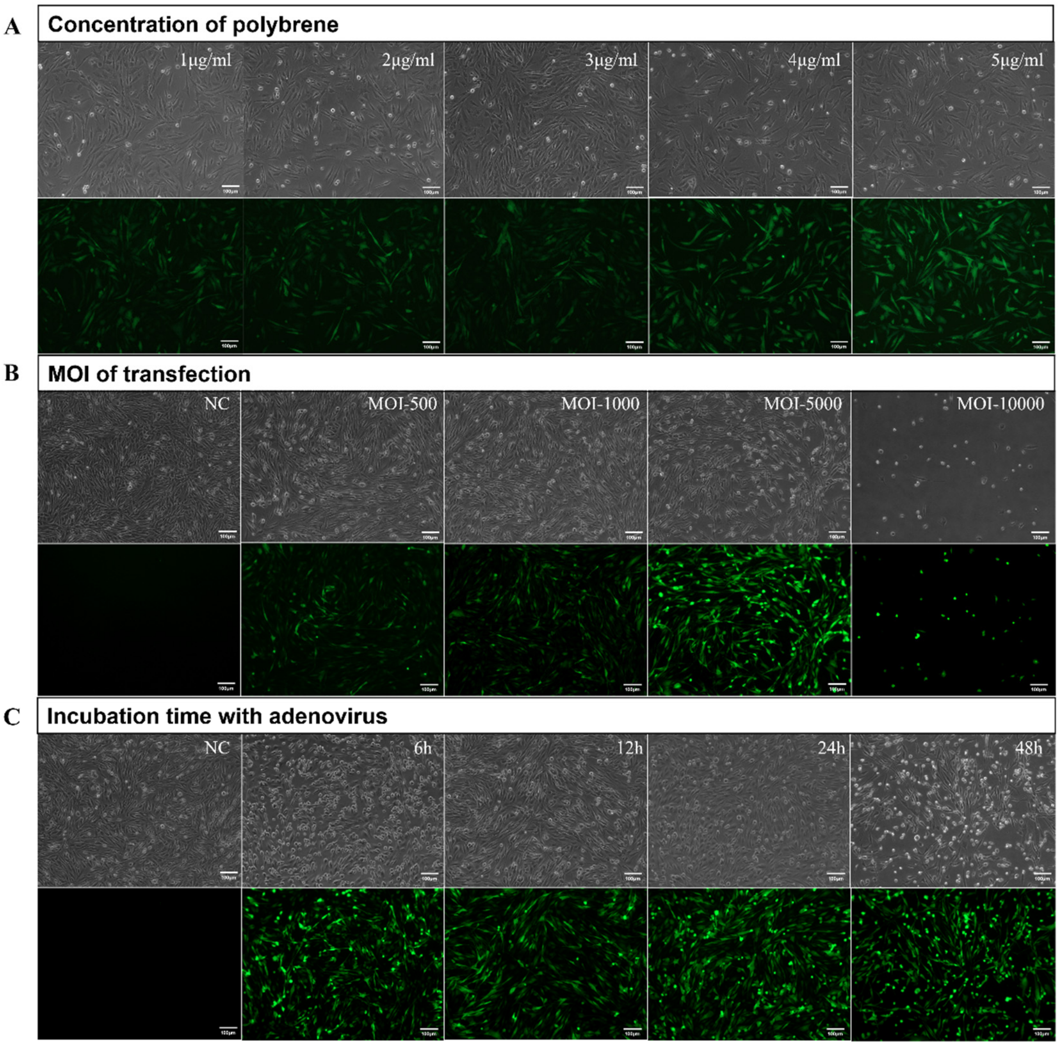

Figure S1. Optimization of conditions for adenoviral vector transfection of DF1. (**A**) Images of DF1 cell adenoviral vector transfected with polybrene added in different concentration. (**B**) Images of DF-1 cell transfected with adenoviral vector for 48 hours in different MOI. (**C**) Images of DF-1 cell transfected with adenoviral vector for different time with MOI=5000. upper images were taken in phase contrast mode and lower were fluorescence images.

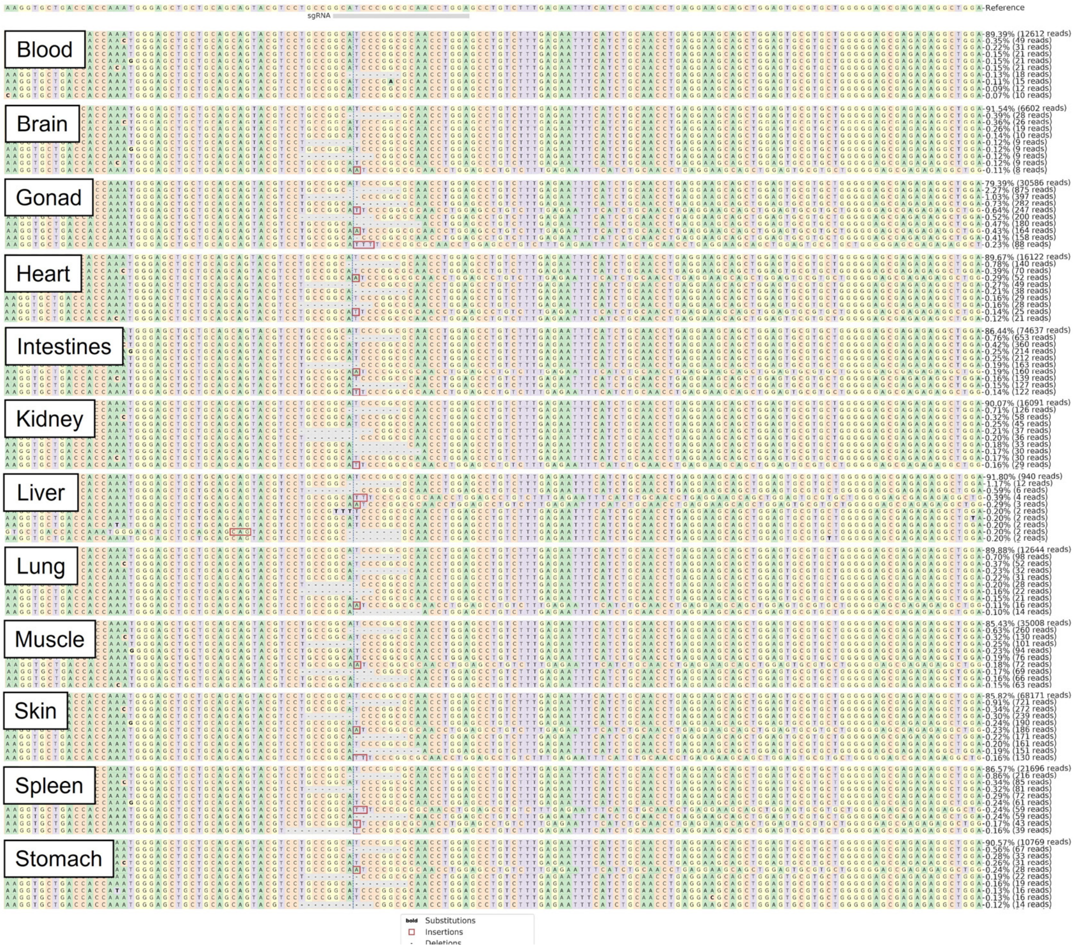

Figure S2. Visualization of the distribution of identified alleles around the cleavage site for the pX459-sgRNA-E2-sgRNA2 in tissues. Nucleotides are indicated by unique colors (A = green; C = red; G = yellow; T = purple). Top 10 of modification patterns were listed in every tissue. Substitutions are shown in bold font. Red rectangles highlight inserted sequences. Horizontal dashed lines indicate deleted sequences. The vertical dashed line indicates the predicted cleavage site.
